# Supplementary material for: Personal relative deprivation and pro-environmental intentions
Source: PLoS One. 2021 Nov 18;16(11):e0259711. doi: 10.1371/journal.pone.0259711 (PMC8601418; doi:10.1371/journal.pone.0259711)
Supplement: S1 Appendix — (PDF) [file pone.0259711.s004.pdf]

Study Materials

Below we reproduce the instructions and items for each component of the studies. For the PRD and ENV scales, the sequence of the items matches their numbering in the data files (e.g., the first PRD item is labelled PRD_1 in the data files).

CONSENT: For Studies 2 and 3, participants had to answer yes/no to the following questions:

1. I confirm that I have read and understood the information about the study.
2. I understand that my participation is voluntary and that I am free to withdraw at any time, without giving any reason.
3. I understand that all personal information will remain confidential and that all efforts will be made to ensure I cannot be identified (except as might be required by law).
4. I understand that following open data recommendations the data and findings obtained in this study may be shared with the research community, for example, by uploading the anonymized data file onto an online data depositor
5. I confirm that I am at least 18 years of age.
6. I agree to take part in the above study.

PRD:

Below you will find various statements. Most likely you will strongly agree with some statements, and strongly disagree with others. Sometimes you may feel more neutral. Read each statement carefully and decide the extent to which you personally agree or disagree with it. Select the response that best corresponds to this judgment using the scales provided.

- I feel deprived when I think about what I have compared to what other people like me have.
- I feel privileged compared to other people like me.
- I feel resentful when I see how prosperous other people like me seem to be.
- When I compare what I have with what others like me have, I realize that I am quite well off.
- I feel dissatisfied with what I have compared to what other people like me have.

These items were listed vertically in a matrix with the response options along the top: Strongly disagree; Disagree; Slightly Disagree; Slightly agree; Agree; Strongly agree, coded 1 to 5.

SSS:

Please read the instruction very carefully and answer the following question.

Think of the ladder below as representing where people stand in the United States [United Kingdom, in Study 2].

At the top of the ladder are the people who are the best off - those who have the most money, the most education and the most respected jobs.

At the bottom are the people who are the worst off - who have the least money, least education, and the least respected jobs or no job.

The higher up you are on this ladder, the closer you are to the people at the very top; the lower you are, the closer you are to the people at the very bottom.

Where would you place yourself on this ladder?

*Please **click** on the rung **where you think you stand** at this time in your life, relative to other people in the United States [United Kingdom, in Study 2].*

(Please select only one rung before advancing)

There followed an illustration of a ladder with 10 rungs; participants could click on a rung to select it.

ENV:

Some people support action on climate change through activities in their personal lives. With this in mind, how likely you are to engage with each of the following activities in the next 12 months?

(If it is not possible for you to engage with an activity, please select 'not applicable')

- Buy environmentally-friendly products
- Conserve water at home (e.g., when cooking or showering)
- Minimize use of heating or air conditioning
- Eat food which is locally-grown or in season
- To check that people are reading, please select "Somewhat unlikely" for this statement
- Buy products with less packaging
- Turn off lights and appliances when not in use
- Avoid or reduce eating meat
- Recycle
- Install products to save energy (e.g., low-energy light bulbs)
- Reduce car travel (e.g., walk, cycle, use public transport)
- Turn off electrical equipment rather than use "standby" mode
- Use car-sharing or car-pooling schemes

These items were listed vertically in a matrix with the response options along the top: Not at all likely (1), Somewhat Unlikely (2), Neither Unlikely Nor Likely (3), Somewhat Likely 4 [sic]; Very Likely (5); Not Applicable. For Studies 2 and 3, the numbers after the verbal response options were removed. Responses were coded from 1 (Not at all likely) to 6 (Not applicable); the latter were ignored when computing the participant mean.

INCOME:

What is your annual household income (before taxes)? [Studies 2 and 3 added: That is, how much does your household make each year, before tax? Please be as accurate as you can.]

- $10,000 or less
- $10,001 - $20,000
- $20,001 -- $30,000

And so on in $10,000 steps until “More than $150,000”. For Study 2, the amounts were in Sterling (e.g., “£10,000 or less”).

EDUCATION:

What is your highest level of educational attainment?

- did not finish high school
- high school graduation
- college graduation
- postgraduate degree

Responses were coded 1 to 4. For Studies 2 and 3, the question was phrased: What is the highest level of education you have completed? For Study 3, the response options were the same as for Study 1 (but with the first letter of each option capitalized); for Study 2 they were:

- No formal qualifications or equivalent (e.g., did not finish school)
- Secondary school qualifications of equivalent (e.g., GCSEs)
- Post-16 education or equivalent (e.g., A-levels, BTEC)
- Bachelors' degree or equivalent (e.g., BA, BSc)
- Master's degree or equivalent (e.g., MA, MSc, MPhil)
- Doctoral degree or equivalent (e.g., PhD)

Responses were coded 1 to 6.

AGE:

What is your age? Study 1: responses were made using a slider ranging from 0 to 100. For Studies 2 and 3, responses were entered via a text box that only accepted numeric input.

GENDER/SEX:

What is your gender?

- Male
- Female
- Non-binary

For Studies 2 and 3 this was replaced with "What is your sex?" with non-binary replaced by "Prefer not to say".

PAST PARTICIPATION

For Studies 2 and 3, past attempts at the survey were probed with the following question:

Which of the following best describes you?

- This is the first time I have attempted this survey
- I have previously started the survey, but did not finish it (e.g., the browser crashed, I lost progress and restarted)
- This is not the first time I have completed this survey; I have previously completed it).

Participant Sampling

Below we describe the process by which the final, analysed samples were obtained.

Study 1: Participants were recruited via Amazon's Mechanical Turk (www.mturk.com); we requested 370 participants from the United States who had been approved for 100 or more Human Intelligence Tasks (HITs; i.e., tasks offered via the platform) and who had an approval rate of 91% or above. The survey software was set to block participation from IP addresses that had already completed the survey. We excluded respondents who met one or more of the following criteria: did not provide responses to all questions; selected more than one rung of the SSS ladder; had an IP address that occurred earlier in the data file (in the case of overlapping time-stamps, both instances were excluded); indicated an age under 18 or greater than 100; selected the wrong response for the attention check. The data file contained 383 entries. Of these, 367 answered all questions. Of these, 38 selected something other than 1 rung of the SSS ladder. Of the remaining 329 participants, 18 were flagged as IP duplicates; of the remainder, no participant indicated an age under 18 or over 100, but 3 failed the attention check, leaving the final sample of 308.

Studies 2 and 3: the participant sampling proceeded was as described in the pre-registrations. Participants were recruited via Prolific (www.prolific.ac). For each study, we requested 440 participants aged 18-100 who were UK-resident (Study 2) or USA-resident (Study 3), not currently students, whose first language was English, whose approval rating on the platform was 98% or greater, using a desktop computer, and (for Study 3) who had not participated in Study 2. The surveys started with a "captcha" to help screen-out automated responding. The software was also set to screen out participants who were using a mobile device, or who were coming from outside the UK (Study 2) or USA (Study 3), or who answered "no" to one or more consent questions; such participants were asked to return the job to Prolific. The survey software was set to block participation from IP addresses that had already completed the survey (this should include those who have previously been redirected and asked to return the survey, but this may not work in practice). We rejected submissions from participants who indicated an age under 18 or greater than 100, or who did not provide complete data (i.e., did not finish the task); the recruitment platform replaced them until 440 people had been approved. We then excluded respondents who had an IP address or Prolific ID that occurred earlier in the data file or in an earlier data file (e.g., in Study 1; in the case of overlapping time-stamps, both instances were excluded), selected the wrong response for the attention check, or indicated that this was not their first attempt at the survey. For Study 2, we also excluded participants whose IP address was detected as being from outside the UK by the maxmind function from the rgeolocate package version 1.3.1 [1]. (This approach was dropped from the pre-registered sampling plan for Study 3 because we suspect that it is prone to false-positives, and preferred to rely on the self-reported country of residence and Qualtrics' IP-based geolocation screening.)

For Study 2: there were 481 entries in the data file. Of these, 41 did not provide complete data. No-one selected something other than 1 rung of the SSS ladder (as described in the main text, the software was set to prevent this; the fact that participants could do so in Study 1 was a glitch). After excluding potential duplicates there were 432 participants; of these, 7 self-reported possible past participation. Of the remaining 425, 14 were flagged as potentially outside the UK. None of the remaining participants indicated an age less than 18 or greater than 100, but 2 failed the attention check, leaving the final sample of 409.

For Study 3: there were 493 entries in the data file. Of these, 53 did not provide complete data. None of the remaining 440 selected something other than 1 rung of the SSS ladder (again, the

software was set to prevent this), but 12 were flagged as potential duplicates. Of the remaining 428, 3 self-reported possible past participation. None of the remaining 425 indicated an age less than 18 or greater than 100, but 5 failed the attention check, leaving the final sample of 423.

References

1. Keyes O, Schmidt D, Robinson D, Davis C, Rudis B, Maxmind Inc, et al. rgeolocate: IP address geolocation. 2020. Available: https://cran.r-project.org/package=rgeolocate

Explanation of Data Files

S1 Data.csv, S2 Data.csv, and S3 Data.csv hold the slightly formatted output from the Qualtrics survey software (e.g., to anonymize the data).

The data files contain some columns which are automatically produced by Qualtrics but which are not informative/considered in our analyses.

The column headings are as follows:

StartDate, EndDate, RecordedDate: timestamps output by survey software. The Start and End times were used when screening for IP duplicates.

Status: Status of response as recorded by Qualtrics.

Progress: percent of the survey completed, as recorded by Qualtrics.

Duration of study session, in seconds, as recorded by Qualtrics.

Finished: Completion of survey, as recorded by Qualtrics. (Can be misleading because it is listed as "0" if the participant closes the browser on the debriefing page.)

ResponseID: unique participant identifier assigned by survey software.

DistributionChannel: As output by Qualtrics. (All Studies were distributed using an anonymous survey link via MTurk or Prolific).

UserLanguage: User language, as recorded by Qualtrics.

(For Studies 2 and 3): Consent_1 to Consent_6. Responses to consent questions (see Materials); 1 = no, 2 = yes. Only participants who answered "yes" to all questions were able to progress.

PRD_1 to PRD_5: Responses to the PRD scale items. See Materials for details.

SSS_1 to SSS_10: rungs on the SSS ladder. 0 means the rung was not selected, 1 means it was.

ENV_1 to ENV_13: Responses to the pro-environmental intentions items (including the attention check, ENV_5). See Materials for details.

educ: level of education. See Materials for details.

income: annual household income. See Materials for details.

age: age, in years

sex: sex (or, for Study 1, gender), where 1 = male, 2 = female, 3 = other (Study 1) or prefer not to say (Studies 2 and 3). These values were recoded as -0.5, 0.5, and 0 by the analysis script.

(For Studies 2 and 3): pastatt: Response to question about past participation; 1 = This is the first time I have completed this survey; 2 = I have previously started the survey, but did not finish it (e.g., the browser crashed, I lost progress and restarted); 3 = This is not the first time I have completed this survey; I have previously completed it.

taskorder: Order of the PRD, ENV and SSS measures.

duplicate: IP address or Prolific ID has appeared with an earlier or overlapping timestamp; 0 = no, 1 = yes

(Study 2 only): country_name: Country based on IP screening via rgeolocate package.

(Study 1 only): state: current US state of residence
