# Supplementary material for: Personal relative deprivation and pro-environmental intentions
Source: PLoS One. 2021 Nov 18;16(11):e0259711. doi: 10.1371/journal.pone.0259711 (PMC8601418; doi:10.1371/journal.pone.0259711)
Supplement: Attachment — Submitted filename: Response to Reviewers.docx [file pone.0259711.s009.docx]

**Response to Reviewers**

Below we reproduce the Reviewers’ comments in full and describe how we have responded to each one.

**Reviewer #1:**

*Thank you for providing me with the opportunity to review this paper for PLOS One.
The authors explore an interesting question in their studies, that being, the extent to which people’s motivations to engage in pro-environmental behaviour may be related to their sense of personal relative deprivation in comparison to their similar peers.

The authors provide a reasonable (albeit rather slim) theoretical and empirical rationale for their research question, and their empirical work appears to have been conducted in a careful and rigorous fashion (and is reported in a refreshingly transparent way).*

*Whilst it is difficult to find fault with the scientific validity of the empirical work, I did feel that the authors could have done a little more to engage with the potential implications of their findings. Obviously, the results were not as clean cut as one might have hoped, or at least the effect sizes were rather small. However, given that the authors do make a general conclusion that PRD seems to be to some extent negatively related to pro-environmental behaviour, it would be good for them to say a bit more about a) why they think this might be, and b) what the potential theoretical implications might be of this being the case, and c) what some of the practical implications of this might be.*

*Re a), the authors suggest in the introduction that possible mediators of the proposed relationship might be greater focus on immediate financial rewards, greater materialism and lower prosociality. Given this, I was slightly surprised to not see any attempt to measure these proposed mediators across any of the reported studies.*

**RESPONSE: We appreciate this point. We planned to look at such mediators in the event that the primary association proved substantial, but given the relatively small correlations that we observed in the current studies, we did not progress to this stage of the research.**

*Re b), is there anything that the findings reported here might tell us about broader theoretical*

*questions, such as the nature of pro-environmental behaviour or indeed the nature of PRD?*

**RESPONSE: As noted in the existing Discussion, we are hesitant to over-speculate, but we have nonetheless added a brief comment about possible broader implications of our findings for circumscribing when PRD will/will not be an important predictor.**

*Re c), if higher levels of PRD might indeed dampen down pro-environmental intentions, then what might this mean for those individuals, organisations or government bodies who seek to increase engagement with pro-environmental behaviour?*

**RESPONSE: We have added a brief note to the Discussion in which we link our findings to this kind of practical concern.**

*At present, the ending of the paper simply states limitations of the study. Stating limitations is important and useful. However, I think the paper would benefit greatly from the addition of a final paragraph that brings things back to considering the wider research problem that was laid out at the start of the introduction, and that considers issues a, b and c outlined above.*

**RESPONSE: Thanks – we have added two paragraphs to the Discussion in which we outline some thoughts about the foregoing points.**

*As a very minor point, there appeared to be a typo on page 16 (“The modest effect is perhaps unsurprising give the complexity and multiply-determined nature of environmental attitudes and behavioural intentions”)*

**RESPONSE: Thanks for spotting this; we’ve corrected it.**

*Additionally, in the discussion the authors state that “we have not examined the possibility that the effects of relative deprivation or social status might vary by gender”. Surely the authors have the data at their disposal to test this though?*

**RESPONSE: We do, but it wasn’t in our pre-registered analysis plan so we are leaving it as a possible direction for the future (interested readers would be welcome to explore the issue with our data as a starting point).**

**Reviewer #2:**

*The manuscript “Personal relative deprivation and pro-environmental intentions” examines the correlation between personal relative deprivation and pro-environmental intentions. In three studies, the authors find a weak but significant effect.*

*The topic is very interesting, and I believe that much research is needed in this domain. Moreover, I think the sample sizes are very nice. However, while I think the aim of the manuscript is important, I have some concerns about the contribution of the manuscript, as I outlined below:*

*1. The paper lacks a theoretical framework. The authors should broaden the theoretical framework and better explain why PRD should affect pro-environmental intentions and ultimately behavior (although it is important to note that the current studies only measured intentions and not behavior).*

**RESPONSE: We have expanded the Introduction to give a clearer sense of our theoretical approach/motivation, and to acknowledge that we examined intentions rather than behaviours (a point which we return to in the Discussion). Note that this aspect of the revision involves expansion/clarification of the material we had previously included – we have not “magicked up” a theoretical rationale that wasn’t there before. Of course, some readers might not find the rationale compelling; unfortunately, there’s not much we can do about that at this point.**

*2. Relatedly, the authors talk about pro-environmental behavior but only measure intentions. Therefore, the gap between intentions and behaviors should be discussed both in the Introduction and Discussion sections.*

**RESPONSE: We now acknowledge the limitations of focusing on intentions in the Introduction and Discussion, and point the reader to a recent review of the intention-behaviour gap**.

*3. The results are very weak. While I think that even null results might be informative (and thus, even when hypotheses are not confirmed, the data could be informative and important), I am not sure what the current paper’s theoretical and practical contribution is.*

**RESPONSE: This is a very fair point. We’re not sure either, but our understanding is that this is not a criterion for publication in this journal. Nonetheless, as noted in the response to Reviewer #1, as noted above we have expanded the Introduction and Discussion to give some additional sense of the contribution of the work.**

*4. Why Study 1 was exploratory? Exploratory in what sense?*

**RESPONSE: Sorry if this wasn’t clear. We think the term “exploratory” is quite widely used to indicate studies for which the sampling and analysis plan is not wholly specified in advanced. We have clarified this with an appropriate reference (Wagenmakers et al., 2012) when describing Study 1.**

*5. Table 1 seems redundant. The Min, Max, and mean values of the variables are not very informative. I think it could be moved to the supplemented materials, while the Cronbach alphas could be indicated in the method section*

**RESPONSE: Thanks for this suggestion. After trying different approaches, we have left the table as it was; readers who prefer to skim over it are welcome to do so.**

*2. The manuscript includes some typos (e.g., PRDS instead of PRD in several locations). Please correct*

**RESPONSE: Thanks – we have checked for, and corrected, typos.**

*I hope the authors will find these comments useful as they continue with this interesting project*

**RESPONSE: They were – thank you.**

**Reviewer #3:**

*This manuscript examines the impact of personal relative deprivation (PRD) on willingness to act in ways to protect the natural environment. It compares the impact of PRD to other frequently investigated indicators of social (class) standing, and thus offers insight into how perceptions rather than more objective indicators affect behavioral intentions. It seems to meet the criteria for publication in Plos One (*[*https://journals.plos.org/plosone/s/criteria-for-publication*](https://journals.plos.org/plosone/s/criteria-for-publication)*), e.g., the research is original, not published elsewhere, meets high methodological, statistical, and (largely) ethical standards, is written clearly. Yet I think that there are ways to improve the manuscript.

Despite the authors’ commendable endeavors to secure results from three samples (two of which were registered), the manuscript could have more forcefully communicated why studying PRD in relationship to environmental behavior intentions was important. It seemed that they just tacked an additional variable on to a long list of other status indicators known to be associated with such behaviors. While the authors tell us how PRD is related to materialism, prosociality, and so forth, they simply conclude that it “might be associated with reduced pro-environmental behavioral intentions.” How will knowing of this association affect expectations for such behavior? Could the argument be couched so that a direction of the association be indicated? Given the other more objective indicators of social standing, should PRD matter more or less? In other words, the justification for the study and the theoretical argument shaping the analyses struck me as thin and could certainly be bolstered.*

**RESPONSE: As noted in our response to Reviewer #2, we have expanded the Introduction to give a clearer sense of the rationale for our work – without straying into excessive/post hoc theorizing.**

*Methodologically the research seems sound. I was, however, concerned that participation in Studies 2 and 3 was not fully voluntary as “all questions required a response before the participant could progress.” Typically, study volunteers are allowed to skip questions thereby upholding their right to continue or discontinue with the study. (I recognize that the studies underwent ethical review, but this requirement would have been disallowed by my university’s Institutional Review Board.)*

**RESPONSE: Participation was voluntary in the sense that people were free to quit at any time, so they don’t have to answer all the questions if they don’t want to. We appreciate that different review boards have different approaches to this, but for our departments this is common.**

*I admit that I longed for a regression table rather than the figures of confidence intervals as I find interpreting coefficients easier.*

**RESPONSE: We are sorry to hear that. We note that the regression tables are provided in the Supplementary Materials (and we’ve clarified this in the main text) so they should be only a click away (via hyperlinks in the text) for the interested reader.**

*The authors note on page 9, line 231 that conducting a fixed effects meta-analysis was a mistake, but do not explain why it was a “mistake” except to say a random effects model makes more sense. Is such a passage necessary?*

**RESPONSE: We have deleted the exposition and now just say that we pre-registered our intention to conduct a fixed effects analysis as well as the random effects analysis.**

*I had hoped that the discussion might do more than simply recount the findings – which demonstrate that PRD really had little effect, especially in the context of the other demographic and social standing variables. Plos One offers the possibility of publishing such null effects, but I nonetheless wondered about the meaning of this pattern of findings in terms of understanding what counts in producing pro-environmental behaviors. The authors do note that other variables could be considered. Given that much previous research has considered many other factors (social, cognitive, emotional), I still wonder about the contribution even though such concerns seem outside of the given review criteria.*

**RESPONSE: We share this concern, but our feeling was that other researchers might (like us) think the question was worth asking, and that it’s best for our somewhat inconclusive results not to sit in a “file drawer”. As noted in our response to Reviewer #1, we have added material to the Discussion that offers some consideration of the implications of our results – without wishing to get carried away with unwarranted speculation.**
